# Genome-wide analysis of interactions between ATP-dependent chromatin remodeling and histone modifications

**DOI:** 10.1186/1471-2164-10-304

**Published:** 2009-07-08

**Authors:** Zhiming Dai, Xianhua Dai, Qian Xiang, Jihua Feng, Jiang Wang, Yangyang Deng, Caisheng He

**Affiliations:** 1 Electronic Department, Sun Yat-Sen University, Guangzhou, PR China

## Abstract

**Background:**

ATP-dependent chromatin remodeling and the covalent modification of histones play central roles in determining chromatin structure and function. Although several specific interactions between these two activities have been elaborated, the global landscape remains to be elucidated.

**Results:**

In this paper, we have developed a computational method to generate the first genome-wide landscape of interactions between ATP-dependent chromatin remodeling and the covalent modification of histones in *Saccharomyces cerevisiae*. Our method succeeds in identifying known interactions and uncovers many previously unknown interactions between these two activities. Analysis of the genome-wide picture revealed that transcription-related modifications tend to interact with more chromatin remodelers. Our results also demonstrate that most chromatin remodeling-modification interactions act via interactions of remodelers with both histone-modifying enzymes and histone residues. We also found that the co-occurrence of both modification and remodeling has significantly different influences on multiple gene features (e.g. nucleosome occupancy) compared with the presence of either one.

**Conclusion:**

We gave the first genome-wide picture of ATP-dependent chromatin remodeling-histone modification interactions. We also revealed how these two activities work together to regulate chromatin structure and function. Our results suggest that distinct strategies for regulating chromatin activity are selectively employed by genes with different properties.

## Background

The nucleosome is the fundamental repeating unit of eukaryotic chromatin. DNA wrapped in a nucleosome is less accessible than linker DNA, nucleosome positioning thus plays an important role in diverse cellular processes that rely on access to genomic DNA. In general, cells devise two main schemes to regulate nucleosomal influences on these cellular processes. One way is through chromatin remodeling, utilizing ATP hydrolysis to alter the histone-DNA contact, often resulting in changed nucleosomal location [1]. As a consequence, ATP-dependent chromatin remodeling changes the accessibility of nucleosomal DNA. For example, one function of the remodelling enzyme Isw2 is to prevent transcription initiation from cryptic sites by repositioning nucleosomes [2]. Another way involves covalent modification of histone tails and globular domains, including acetylation, methylation, phosphorylation, sumoylation, ubiquitination, and adenosine-diphosphate ribosylation. Modifications not only establish chromatin environments for recruitment of nonhistone proteins, but also affect the contacts between different histones in adjacent nucleosomes or the interactions of histones with DNA [3]. For instance, acetylation can neutralize the positive charge of the lysine, acetylated histone tails are thus thought to associate more loosely with nucleosomal DNA than unmodified histone tails [4].

It has become clear that there is a connection between ATP-dependent chromatin remodeling and covalent histone modifications [5,6]. Chromatin remodelers bind modified histone residues via specific domains. SNF2-type chromatin remodelers have bromodomains for binding acetylated lysines [7]. CHD-type chromatin remodelers harbor chromodomains that bind methylated lysines [8]. On the other hand, experimental evidence has showed direct crosstalk between chromatin remodeling complexes and histone-modifying complexes. For example, Isw1, one remodelling enzyme, physically interacts with the histone deacetylase activity of the Sin3A/Rpd3 complex [9].

Although several particular interactions between chromatin remodeling and histone modifications have been elaborated [10], the full landscape remains to be elucidated. In addition, it is less clear whether cooperativity between remodeling and modification has different effects on genome-wide properties versus independent remodeling or modification. In this study, we have developed a computational approach to derive the first genome-wide landscape of interactions between ATP-dependent chromatin remodeling and histone modifications in budding yeast *Saccharomyces cerevisiae*. Our method succeeds in identifying known interactions and uncovers many previously unknown interactions between these two activities. Further insights into this landscape showed that transcription-related modifications tend to work with more ATP-dependent chromatin remodelers. We found that certain chromatin remodelers are linked to a great number of histone modifications. Our results suggest that remodelers interact with both histone-modifying enzymes and histone residues. We also explored the effects of cooperativity between remodeling and modification versus independent remodeling or modification on gene properties. We defined three gene cohorts of independent modification, independent remodeling, and both modification and remodeling. We analyzed these gene classes and showed how they differ in multiple gene properties, including nucleosome occupancy, H2A.Z occupancy, binding site locations and numbers, RNA Polymerase II (RNAP II) occupancy, histone turnover, and gene activity.

## Results

### Construction of the landscape

To construct the landscape of interactions between ATP-dependent chromatin remodeling and histone modifications in budding yeast, we used two data sets that provide a genome-wide measurement of enrichment levels of 25 histone modifications [11,12] and changes in gene expression accompanying the perturbation (mutation or deletion) of 33 ATP-dependent chromatin remodelers [13]. We first identified cohort of genes for each modification and chromatin remodeler. Genes belong to one modification cohort if they display significantly high levels (Z score > 1.64, *P value *< 0.05) of the corresponding modification at promoters (see Additional file 1). In this way, we obtained 25 sets of modification cohorts. The second data set is from a previously assembled expression compendium of chromatin modifiers [13]. Genome-wide changes in gene expression were measured when various chromatin modifiers were deleted or mutated. We restricted the analysis to expression profiles for perturbation of ATP-dependent chromatin remodelers. This resulted in a refined data set consisting of 33 expression profiles. Considering that there are dual remodelers acting as both activators and repressors, we determined both positively and negatively regulated cohorts for each chromatin remodeler. Genes belong to positively (negatively) regulated cohort for one chromatin remodeler if they display significantly decreased (increased) changes (*Z *score < -1.64 and *P *value < 0.05 for decreased changes, *Z *score > 1.64 and *P *value < 0.05 for increased changes) in gene expression accompanying the perturbation of the corresponding remodeler (see Additional file 1). The details of data process in this paper are described in Methods section below.

Having identified the cohort for each modification and remodeler, we next determined whether the remodeler interacts with the modification or not using two criteria. First, we employed modification cohorts and the expression compendium of chromatin remodelers to identify the interactions. If the remodeler works in concert with the modification to regulate chromatin activities of a subset of genes, its perturbation should cause a differential change in expression of modification cohort genes because gene expression is linked with chromatin regulation. As in a previous study [13], we used the Kolmogorov-Smirnov (K-S) statistical test. The K-S *P *value provides a measure of the discrepancy in the distribution of gene expression values between the modification cohort and the rest of the genes. The K-S score indicates both the direction and significance of the discrepancy between the two distributions. A positive sign of the K-S score indicates positive regulation by the remodeler (i.e. the modification cohort genes tend to have lower expression values than the rest of the genes accompanying the perturbation of the remodeler), whereas a negative sign implies negative regulation. Second, we utilized remodeling cohorts and the modification data to detect the interactions between chromatin remodeling and modifications. If the chromatin remodeling interacts with the modification, its cohort should show significantly higher levels of the corresponding modification at promoters. We used the Mann-Whitney U-test to evaluate the difference in the medians of modification levels between the positively (negatively) regulated cohort and the rest of the genes. To avoid confusion in the following analysis, the *P *value was set as 1 if the positively (negatively) regulated cohort genes show significantly lower modification levels than the rest of the genes. For each remodeler-modification pair, either positively or negatively regulated cohort was selected according to the sign of its K-S score determined above. We use this selection of remodeling cohort for each pair in the following analysis if there is no other statement.

**Figure 1:**
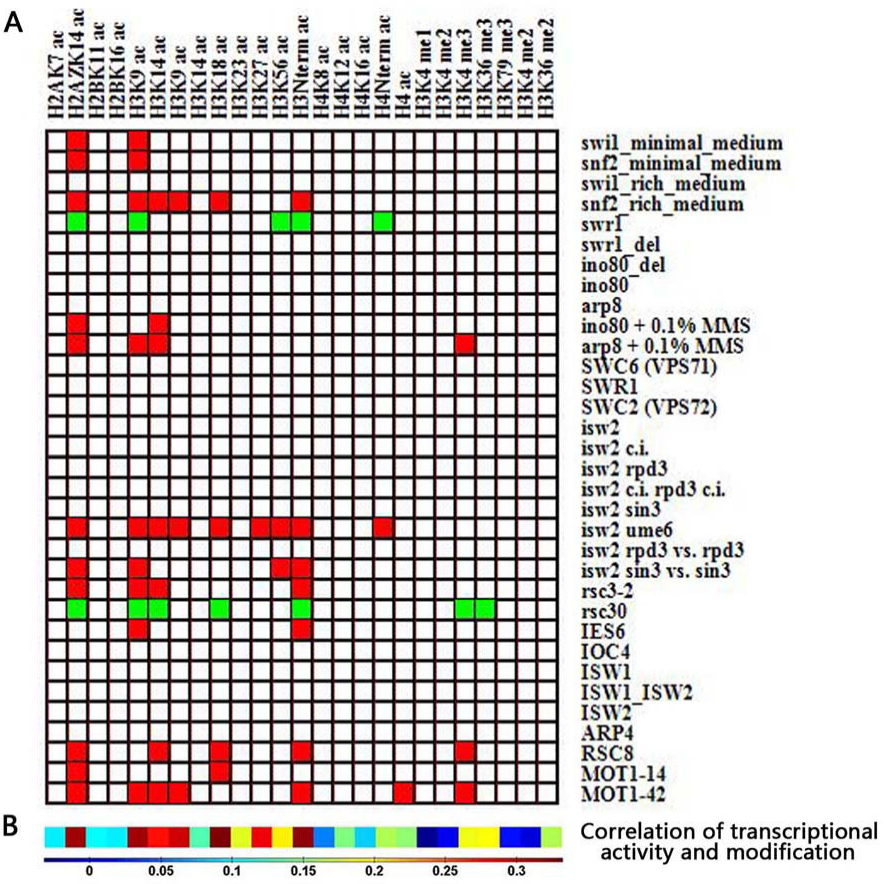
**The interactions between ATP-dependent chromatin remodeling and covalent modification of histones**. (A) Rows represent ATP-dependent chromatin remodelers, and columns represent histone modifications. Ac represents acetylation, while me indicates methylation (e.g. me2 indicates dimethylation). For each remodeler-modification pair, if it satisfies *P *< 0.05 in both statistical tests, it was colored red (positive regulation by the remodeler) or green (negative regulation by the remodeler), otherwise it was colored white. (B) The correlation between transcriptional activity [26] and modification enrichment at promoters for the histone modification indicated in each column. Colors indicate Pearson correlation coefficients.

We carried out the above two statistical tests for all chromatin remodeler-modification pairs and generated 61 significant pairs with *P *< 0.05 (after Bonferroni correction for multiple testing) in both tests (Figure 1, see Additional file 2). However, the resulting interactions may be biased by transcriptional effect, as some modifications and remodeling both required for high gene activity may appear correlated, for no specific reason. To assay whether the results are biased by transcriptional effect, we performed principal component analysis for each chromatin remodeler-modification pair in terms of its modification and expression levels. We considered the first principal component to be transcription-related and represented each gene in the first principal component space. For each chromatin remodeler-modification pair, we calculated Pearson correlation coefficient between the above representation of each gene and its transcription rate. If the identified 61 significant pairs are biased by transcriptional effect, they should have significantly higher correlation coefficients. However, we found that there is no significant difference in the resulting correlation coefficients between the identified 61 pairs and the other pairs (*P *= 0.11, t-test). Similar result could be reproduced when we represented genes in the second principal component space.

A recent study has measured occupancy at every yeast promoter region for seven chromatin remodelers (Isw1a, Isw1b, Isw2, Swi/Snf, Rsc, Ino80, and Swr-c) [14], yielding opportunity for examining direct targets of the remodeling activities. Using this small dataset along with histone modification data, we carried out similar analysis to derive the interactions between chromatin remodeling and histone modifications. Genes belong to targets of one remodeler (i.e. cohort) if they display significantly high occupancy levels (*Z *score > 1.64, *P *value < 0.05) of the corresponding remodeler at promoters (see Additional file 1). A remodeler-modification pair is determined to be associated if the modification cohort genes exhibit significantly higher occupancy levels of the corresponding remodeler and the remodeling cohort genes have significantly higher corresponding modification levels. Our analysis generated 13 significant pairs with *P *< 0.05 (after Bonferroni correction for multiple testing) in both Mann-Whitney U-tests (see Additional file 3), 8 of which are included in the 61 significant pairs generated above. Moreover, for 12 of the 13 significant pairs, the modification cohort genes exhibit significantly different expression levels (*P *< 0.01, K-S test) accompanying the perturbation of the corresponding remodeler and the remodeling cohort genes have significantly higher corresponding modification levels (*P *< 0.01, Mann-Whitney U-test). These results show that our method is robust to the choice of dataset.

Based on the 61 identified pairs above, we provided the first global picture of interactions between ATP-dependent chromatin remodeling and histone modifications in a eukaryote (Figure 1). In the following analysis, we focused on the 61 significant remodeler-modification pairs.

### Specificities of the landscape

The landscape demonstrates the characteristic interactions between ATP-dependent chromatin remodeling and histone modifications in regulating chromatin activity. We found that there is selectivity of histone modifications and chromatin remodelers involved in the interactions: 12 of the 25 histone modifications show interactions with chromatin remodelers, and 14 of the 33 remodelers are connected with histone modifications (Figure 1). Furthermore, some specific remodelers work in concert with more histone modifications than the other remodelers. There are six remodelers (Snf2, Swr1, Isw2 & Ume6, Rsc30, Rsc8, and Mot1) that interact with five or more histone modifications. Interestingly, these six remodelers show a preference for histone acetylation. We next investigated whether there is known experimental evidence in previous studies supporting our identified interactions.

**Figure 2:**
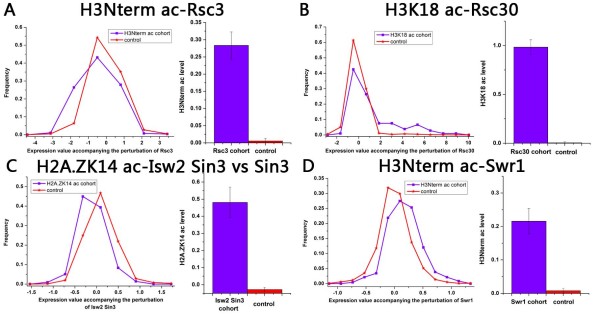
**Selected significant modification-remodeling pairs**. (A-D) Distributions of expression levels (log2 transformed) accompanying the perturbation of the remodeler are presented for the modification cohort and the control group (rest of the genes). Modification levels at promoters are also shown for the remodeling cohort and the control group. Ac represents acetylation, while me indicates methylation (e.g. me3 indicates trimethylation). Error bars were calculated by bootstrapping.

Rsc3, Rsc8 and Rsc30 are components of the RSC chromatin remodeling complex which contains almost half of the known bromodomains in the yeast genome for binding acetylated lysines [15]. A recent study has demonstrated that the ATP-dependent remodelling complex RSC shows a striking preference for H3 but not H4 acetylated chromatin [5]. It has also been reported that H3K14 (i.e. histone H3 lysine 14) acetylation acts to increase recruitment of the RSC to nucleosomes [16]. Our results are consistent with these observations: the three components interact with H3 acetylation, but not H4 acetylation. Rsc3 interacts with H3K9, H3K14, and H3Nterm acetylation, Rsc30 is associated with H3K9, H3K14, H3K18, and H3Nterm acetylation, and Rsc8 is connected with H3K14, H3K18, and H3Nterm acetylation. In addition to these known interactions, our results show that Rsc3 interacts with H2A.ZK14 acetylation, Rsc30 is associated with H2A.ZK14 acetylation, H3K4 and H3K36 trimethylation, and Rsc8 is connected with H2A.ZK14 acetylation and H3K4 trimethylation. Interestingly, our results demonstrate that Rsc3 and Rsc30 regulate the associated modification cohort in distinct ways (positive and negative regulation, respectively, Figure 2A, B), consistent with experimental evidence that they have different roles in regulation, although they interact physically [17].

Swr1 is Swi2/Snf2-related ATPase that is the structural component of the SWR1 complex. Bdf1, a member of SWR1 complex, contains two bromodomains that recruit SWR1 to acetylated histones [18]. Consistent with this, Swr1 interacts with H2A.ZK14, H3K9, H3K56, H3Nterm, and H4Nterm acetylation. Unlike most other remodelers, Swr1 regulates the associated modification cohort negatively (Figure 2D). We found that the Swr-c cohort genes (defined by the remodeler occupancy data) show lower transcription rates than the other genes (*P *< 10^-6^, Mann-Whitney U-test), although SWR1 is required for deposition of histone H2A.Z which is linked to gene activation [19].

Snf2 and Swi1 belong to the SWI/SNF chromatin remodeling complex that contains bromodomains. Specifically, it has been shown that the Swi2/Snf2 bromodomain has a higher affinity for acetylated H3K9 and H3K14 peptide compared with unmodified H3 peptide [20]. Our results not only reproduce this observation, but also show interactions of Snf2 with H2A.ZK14, H3K18, and H3Nterm acetylation in rich media. However, Snf2 and Swi1 interact with only H2A.ZK14 and H3K9 acetylation in minimal media. These results suggest that their regulatory manner differs between the two growth conditions.

Mot1, a member of the Snf2/Swi2 protein family of ATPases, functions by removing TATA-binding protein (TBP) from DNA. In addition, Mot1 is required for nucleosome remodeling independently of TBP recruitment [21]. To our knowledge, there is no experiment exploring the interactions between Mot1 and histone modifications. We showed that different mutations of Mot1 (*mot-14 *and *mot-42*) affect its interactions with different histone modifications. Together, Mot1 interacts with H2A.ZK14, H3K9, H3K14, H3K18, H3Nterm, H4 acetylation, and H3K4 trimethylation. Its preference for histone acetylation may be due to the bromodomains of the Snf2/Swi2 protein family it belongs to [7].

Arp8, Ino80, and Ies6 are subunits of INO80, a chromatin remodeling complex that is involved in regulation of transcription and in DNA damage response [22]. Our results show that Ies6 interacts with H3K9 and H3Nterm acetylation in rich media. In the presence of DNA damage, Ino80 interacts with H2A.ZK14 and H3K14 acetylation, and Arp8 is associated with H2A.ZK14, H3K9, H3K14 acetylation, and H3K4 trimethylation. These results demonstrate that different components of INO80 act to interact with histone modifications in different growth conditions. INO80 and NuA4 histone acetyltransferase complexes share the protein Arp4 [22], which may account for the interaction between INO80 and histone acetylation.

Isw2, one ATP-dependent chromatin remodeling enzyme, is involved in gene activation and repression [23]. We found that there is no interaction between Isw2 alone and histone modification. We thus reasoned that the nine interactions between Isw2 & Ume6 and histone modifications are mainly attributable to transcription factor Ume6. To test this possibility, we examined the transcriptional effect of Ume6 deletion on the nine associated modification cohorts [24]. Seven of the nine cohorts (except H3K56 and H4Nterm acetylation) show significant changes (*P *< 0.05, K-S test) in gene expression upon the deletion of Ume6.

Taken together, we validated our approach to demonstrate that it accurately predicts experimentally determined interactions between ATP-dependent chromatin remodeling and histone modifications (see Additional file 2). In addition to the known interactions, our approach also uncovers many new interactions between these two activities, giving the first global landscape in yeast.

On the other hand, we analyzed the number of remodelers to which each histone modification is linked. Interestingly, only H3, H4 and H2A.Z modifications display interactions with chromatin remodeling. As a previous analysis has indicated that H3–H4 tetramers are ~20 times more stable than H2A-H2B dimers [25], more ATP-dependent chromatin remodelers should be required to modulate H3–H4 tetramers. We also calculated the Pearson correlation coefficient between transcriptional activity [26] and modification enrichment at promoters for each histone modification (Figure 1B). Our result shows that the modifications, which display higher positive correlation with transcriptional activity, tend to work with more ATP-dependent chromatin remodelers. This result suggests that the regulation of chromatin structure at active promoters involves more chromatin remodelers. Recruitment of more chromatin remodelers is expected to make nucleosomes more dynamic. As expected, the cohort promoters for modifications working with at least four chromatin remodelers have significantly higher rates of histone H3 turnover [27] than those for modifications working with at most one chromatin remodeler (*P *< 10^-92^, Mann-Whitney U-test).

### Mechanisms of the interactions between the two activities

We sought to understand the mechanisms of how ATP-dependent chromatin remodeling interacts with histone modifications. One possible mechanism is through interactions between chromatin remodeling complexes and histone-modifying complexes [28,29]. Another mechanism involves links between histone residues and remodelers [30]. We examined the genome-wide prevalence of these two mechanisms. To test the first possibility, we first determined histone-modifying enzymes for each histone modification using modification cohorts of genes and expression profiles accompanying the perturbation of histone acetyltransferases (HATs) and methyltransferases (HMTs) [13]. If the histone-modifying enzyme directs the histone modification, its perturbation should cause a differential change in expression of modification cohort genes. As the first strategy above for detecting remodeling-modification interaction, we used the K-S statistical test to derive histone-modifying enzymes for each histone modification (*P *< 0.05 and positive regulation, after Bonferroni correction for multiple testing, see Additional file 2). For each remodeling-modification interaction identified above, we determined interaction state between chromatin remodeler and histone-modifying enzymes of the histone modification using a general repository of experimentally determined protein-protein interactions [31]. Chromatin remodelers in most remodeling-modification interactions (44 of 61) are shown to interact with at least one histone-modifying enzyme of their connected modifications (see Additional file 2).

We next examined the role of links between histone residues and remodelers in remodeling-modification interactions. Previous studies have measured genome-wide expression when some specific histone residues (H3K4, H3K27, H3N-terminal, H4K8, H4K12, H4K16, and H4N-terminal) were mutated [32,33]. If the histone residue interacts with the remodeler, its mutation should cause a significantly different change in gene expression between the remodeler cohort genes and the rest of the genes. We used the K-S statistical test as above on the remodeler cohorts of genes and expression profiles accompanying the mutation of histone residues. We restricted analysis to the remodeling-modification interactions whose expression profiles accompanying the mutation of the corresponding histone residues are available. Remodeler cohort genes in ~94% of remodeling-modification interactions show significantly different expression changes (*P *< 0.01) accompanying the mutation of the corresponding histone residues (see Additional file 2). This result suggests that histone residues play important roles in remodeling-modification interactions.

Taken together, we showed that most remodeling-modification interactions act via interactions of remodelers with both histone-modifying enzymes and histone residues. The prevalent dual interactions of remodelers also validate our landscape since our remodeling-modification interactions are not trained on interactions of remodelers with both histone-modifying enzymes and histone residues at all. As the two modes of interaction are not mutually exclusive, they might together guarantee the proper interactions between remodeling and modifications.

### The effect of cooperativity between remodeling and modifications

We investigated into the effects of cooperativity between remodeling and modifications versus independent remodeling or modifications on genome-wide properties. To this end, we first identified three gene cohorts of independent modification, independent remodeling, and both modification and remodeling. As mentioned above, genes belong to one modification cohort if they display significantly high levels of the corresponding modification at promoters. Genes belong to regulated cohort for one chromatin remodeler if they display significantly different changes in gene expression accompanying the perturbation of the remodeler. For each of the 61 significant remodeler-modification pairs, the cohort of independent modification includes genes that belong to the corresponding modification cohort but not any remodeling cohort. The cohort of independent remodeling includes genes that belong to the corresponding remodeling cohort but not any modification cohort. The cohort of both modification and remodeling is the intersection between the corresponding modification cohort and the corresponding remodeling cohort. We compiled the three types of cohorts from all identified remodeling-modification interactions, respectively. This yields three sets of genes: modification-independent cohort, remodeling-independent cohort, and modification and remodeling cohort (see Additional file 4). By applying these strict criteria we ensured a low level of false positives for the three distinct cohorts (see Additional file 5). In the following analysis, we focused on the three cohorts.

**Figure 3:**
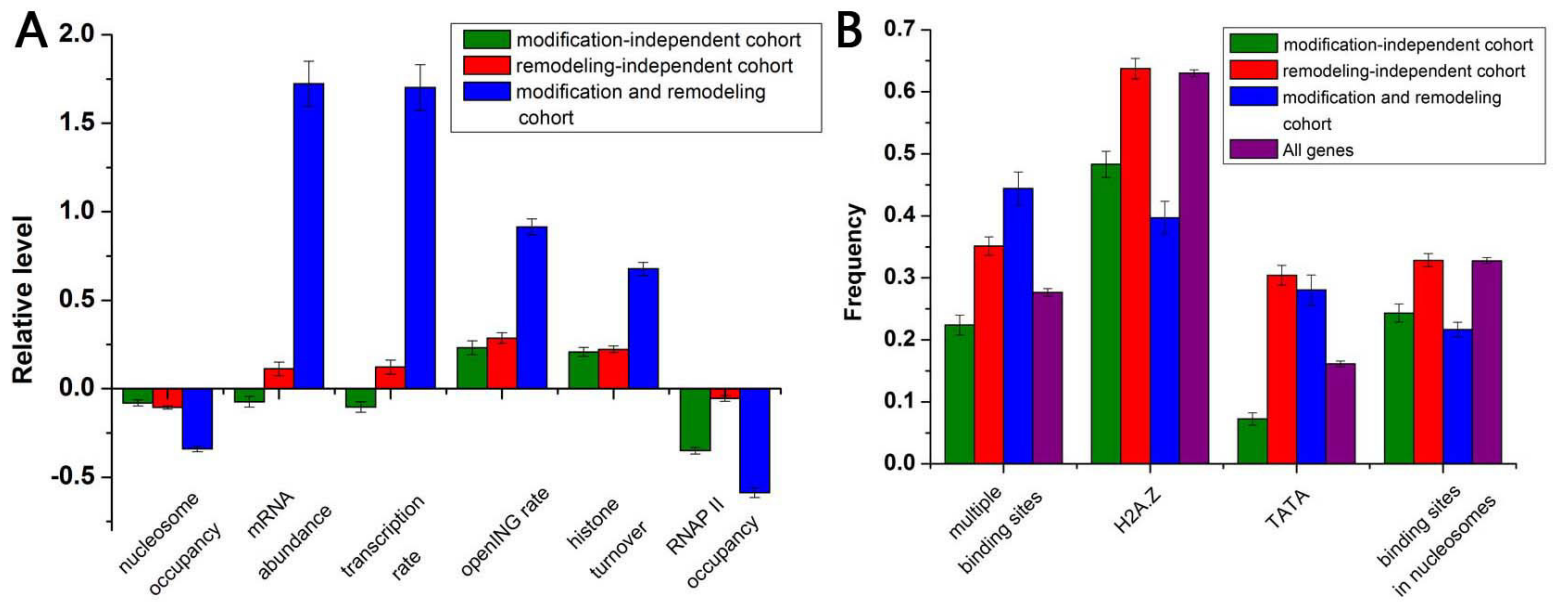
**Gene features that distinguish the three cohorts**. (A) Average values that correspond to nucleosome occupancy [34], transcription rate [26], gene expression level [26], openING rate, the turnover rate of H3 histone [27] and PNAP II occupancy [44] are shown for modification-independent cohort (green), remodeling-independent cohort (red) and modification and remodeling cohort (blue). Values in each property were normalized (nucleosome occupancy and turnover rates were normalized among all promoters, RNAP II occupancy were normalized among all 200 bp upstream regions), such that their means are zero and standard deviations are one. (B) Ratio of transcription factor binding sites [37] localized in nucleosome [34], as well as ratio of promoters with multiple binding sites [37], TATA box [41], and H2A.Z [42] is shown for modification-independent cohort (green), remodeling-independent cohort (red), modification and remodeling cohort (blue) and all genes (purple). Error bars were calculated by bootstrapping.

We first analyzed the three gene cohorts in terms of nucleosome occupancy. Recent studies have measured high-resolution nucleosome occupancy across the yeast genome [34,35]. These valuable data allow for a direct examination of the effect of different activities on nucleosome occupancy. Modification and remodeling cohort promoters have significantly lower nucleosome occupancy [34] than the other two cohorts (Figure 3A). It is known that genomic DNA sequence is an important determinant of nucleosome positioning [36]. However, there is no significant difference in sequence preferences for nucleosomes [34,36] among the three cohorts (data not shown), indicating that the differences in nucleosome occupancy among the three cohorts are not due to the differences in sequence preferences for nucleosomes. These results imply that a combination of ATP-dependent chromatin remodeling and histone modifications causes lower nucleosome occupancy.

We next sought to understand why modification and remodeling cohort promoters have significantly lower nucleosome occupancy than remodeling-independent cohorts. Histone modifications could cooperate with transcription factors (TFs) to regulate DNA-templated processes [10] and TFs could compete with nucleosomes for occupancy along the genome [36]. We asked whether TF binding information contributes to the significant difference in nucleosome occupancy. Indeed, modification and remodeling cohort promoters are highly enriched with TF binding sites [37] compared with the other two cohorts (Figure 3B). Moreover, binding sites are highly localized in linker DNA [34] at modification and remodeling cohort promoters and modification-independent cohort promoters (Figure 3B). We asked whether histone modifications facilitate TF binding or occur as a consequence of TF binding. Experiments on individual TFs and genes revealed that TFs recruit HATs for specific acetylation [38]. The generation of genome-wide expression profiles that correspond to the deletion of various TFs [24] allows us to address this question on a genome scale. If the TF recruits the HAT, its deletion should cause a differential change in expression of the corresponding acetylation cohort as histone acetylation is thought to play an important role in modulation of gene expression [39]. We performed the K-S statistical test on the TF-acetylation associations identified in a previous study [38]. We found only ~47% of pairs whose acetylation cohort show significant change (*P *< 0.01) in gene expression accompanying the deletion of the corresponding TF compared with the rest of the genes (see Additional file 6). One possible explanation for this observation is that multiple TFs may work in a redundant fashion, providing robustness to the regulatory system. Another explanation is that the recruitment of HATs by TFs may not be a universal mechanism of the relationship between TFs and histone acetylation. The study mentioned above has showed that H3K14 and H3K18 acetylation levels decrease in TF hir3 mutant at the YDR224C promoter, and H3K18 acetylation level decreases in TF yml081W mutant at the YDR525W promoter [38]. Our identified significant TF-acetylation pairs include Hir3-H3K18 acetylation and YML081W-H3K18 acetylation pairs. However, we found no significantly different change in expression between the H3K14 acetylation cohort and the rest of the genes when Hir3 was deleted. Indeed, the deletion of Hir3 causes significant decrease (> 2-fold) in expression level of YDR224C. This result indicates that the recruitment of H3K14 acetyltransferases by Hir3 may be specific for individual genes.

Analysis of gene activity revealed that modification and remodeling cohort genes have significantly higher transcription rates and absolute mRNA abundance [26], whereas of the other two cohort genes are comparable to genome-wide levels (Figure 3A). This result is consistent with the general observation that the level of nucleosome occupancy in promoter is inversely proportional to the corresponding gene transcription rate [40]. Furthermore, modification and remodeling cohort promoters have significantly higher rates of histone H3 turnover [27] than those of the other two cohort promoters (Figure 3A). We further analyzed gene activity in various conditions for the three cohorts. We compiled gene expression data from 1,082 published microarray experiments under various cellular conditions. For each gene, we calculated the proportion of experiments in which it displayed significantly up-regulated expression changes, and defined the normalized resulting value as openING rate. The openING rate reflects the general gene activity in various conditions. Modification and remodeling cohort genes also show significantly higher openING rates (Figure 3A), indicating that the higher gene activities of modification and remodeling cohort genes are conserved among various conditions.

We examined whether there is significant difference in other properties among the three cohorts. Modification and remodeling cohort and remodeling-independent cohort promoters are highly enriched with TATA boxes [41], whereas modification-independent cohort promoters are depleted of TATA boxes (Figure 3B). A previous study has shown that nucleosome-inhibited genes tend to have TATA boxes [41]. Chromatin remodeling is thus required to overcome the nucleosomal barrier. H2A.Z nucleosomes help to establish nucleosome-free region (NFR) directly upstream of the transcription start site (TSS) in yeast [42]. Modification and remodeling cohort and modification-independent cohort promoters are depleted of H2A.Z nucleosomes [42] (Figure 3B). Experimental evidence has also shown that H2A.Z nucleosomes tend to appear in hypoacetylation regions [43]. Interestingly, modification and remodeling cohort and modification-independent cohort promoters also have significantly lower RNAP II occupancy [44] at the 200 bp upstream regions (Figure 3A). Our result suggests a potential link between histone modifications and depletion of RNAP II. We further examined whether any of the three cohorts were enriched with genes that are annotated by Gene Ontology (see Additional file 7). Modification-independent cohort is highly enriched with genes that are involved in RNA-related processes. Remodeling-independent cohort genes tend to participate in biogenesis processes. Modification and remodeling cohort genes tend to play housekeeping roles.

## Discussion

ATP-dependent chromatin remodeling and covalent modification of histones play central roles in determining chromatin structure and function. However, how these two activities are coordinated is largely unknown. In this work, we have developed a computational method to establish a genome-wide landscape of connections between ATP-dependent chromatin remodeling and covalent modification of histones in yeast. Our method is able to detect previously known associations and uncovers many new interactions between these two activities. We revealed some key features of the landscape and the mechanisms in which these two activities work together. We also showed the significantly distinct effects of cooperativity between remodeling and modification versus independent remodeling or modification on various gene properties.

Our method is guaranteed to detect remodeling-modification interactions. A previous study has identified interactions between chromatin modifiers and transcription factors by evaluating the discrepancy in expression between the transcription factor cohort and the rest of the genes when the chromatin modifier gene was mutated [13]. Here, we have designed a strategy including two strict criteria to detect remodeling-modification interactions. A remodeler-modification pair is determined to be associated if the modification cohort genes exhibit significantly different expression levels accompanying the perturbation of the corresponding remodeler and the remodeling cohort genes have significantly higher corresponding modification levels. These two complementary filtering processes are very important to ensure a low level of false positives: The former removed 39 pairs that satisfy the latter criteria, and the latter removed 83 pairs that satisfy the former criteria.

Despite its success, our approach has several limitations and represents only a first step towards understanding the interactions between chromatin remodeling and histone modifications. First, additional efforts are needed to distinguish between direct and indirect remodeling-modification interactions. Second, the perturbation of one remodeler might not cause its regulated genes to undergo significant changes in gene expression for two reasons: One is that multiple remodelers might work in a redundant fashion; another is that the mutant of the remodeler might still regulate its target genes properly. As a result, our approach might miss some remodeling-modification interactions.

A sequential series of activities for regulating chromatin is required for transcriptional activation. For example, the ATP-dependent chromatin remodelers recruited by specific histone modifications might initiate a fresh wave of histone modifications [45]. It is well accepted that proteins are recruited to modifications mainly via recognizing and binding modified residues [3]. We found that remodelers not only interact with histone residues, but also interact with histone-modifying enzymes. We reason that the prevalent dual interactions of remodelers guarantee proper transcriptional regulation.

A key finding of this study is that distinct modes for chromatin regulation are associated with distinct gene properties. The co-occurrence of modification and remodeling has significantly different influences on multiple gene features compared with presence of either one. There are two main characteristic functions of modifications: one is to affect the contact between nucleosomes and the interaction between histones and DNA, and the other is the recruitment of nonhistone proteins. Histone modifications that interact with remodeling may recruit more TFs for competing with nucleosomes for occupancy along the genome (Figure 3B). As a result, modification and remodeling cohort genes have significantly lower nucleosome occupancy compared with remodeling-independent cohort genes (Figure 3A). Consequently, modification and remodeling cohort genes have higher gene activity. Moreover, modification and remodeling cohort genes also exhibits higher gene activity in various conditions (Figure 3A). Their lower nucleosome occupancy and mode of chromatin regulation thus seem likely to be conserved among various conditions.

Overall, we gave the first genome-wide picture of interactions between ATP-dependent chromatin remodeling and the covalent modification of histones. We also revealed how these two activities work together and how they regulate chromatin structure and function. The landscape we generated should facilitate the understanding of specific gene regulatory phenomena, such as the mechanisms by which nucleosome positions are coordinated in vivo. The generation of similar experimental datasets in higher eukaryotes will allow us to apply our method to provide further insights into the mechanisms of cooperativity between remodeling and modifications.

## Conclusion

ATP-dependent chromatin remodeling and the covalent modification of histones, play central roles in determining functional state of chromatin. Although several specific interactions between these two activities have been elaborated, the full landscape remains to be elucidated. Here, we have developed a computational method to dissect the genome-wide interactions between ATP-dependent chromatin remodeling and the covalent modification of histones. Our method succeeds in identifying known interactions and uncovers many previously unknown interactions between these two activities in *Saccharomyces cerevisiae*. Analysis of our established genome-wide landscape reveals that transcription-related modifications interact with more ATP-dependent chromatin remodelers. Our results indicate that remodeling-modification interactions are through interactions of remodelers with both histone-modified enzymes and histone residues. Finally, we explored the effects of cooperativity between remodeling and modification versus independent remodeling or modification on gene properties. We defined three gene cohorts of independent modification, independent remodeling, and both modification and remodeling. We analyzed these gene classes and showed how they differ in multiple gene properties.

## Methods

### Histone modifications and chromatin remodelers

Yeast genome sequences were downloaded from the Saccharomyces Genome Database [46]. Histone modification data were taken from ChromatinDB [11], a database of genome-wide histone modification patterns for Saccharomyces cerevisiae. We added the histone modification data from Pokholok et al. [12], resulting in a total of 25 histone modifications. Values have been normalized in the literature. For each promoter (1000 bp upstream of the gene in this study, the upstream region was truncated if it overlapped with neighboring genes), we calculated the average level for each histone modification. We used a compendium of gene expression experiments in which various chromatin modifiers were deleted or mutated [13]. In this work, we restricted the analysis to ATP-dependent chromatin remodelers and histone-modifying enzymes (HATs and HMTs), respectively. For each chromatin remodeler-modification pair, principal component analysis was applied to the data of all genes versus modification and expression levels. Chromatin remodeler occupancy at TSS and UAS, including Isw1a, Isw1b, Isw2, Swi/Snf, Rsc, Ino80, and Swr-c, were taken from Venters et al. [14].

### Nucleosome data

Nucleosome occupancy data were taken from Lee et al. [34], which were normalized among all promoters in that nucleosome occupancy at promoters are lower than that in coding region [34], such that their means are zero and standard deviations are one. For each gene, we calculated the average nucleosome occupancy at promoter. H2A.Z nucleosomes were taken from Albert et al. [42]. To avoid confusion, we restricted the analysis to the 10% most scored H2A.Z nucleosomes in the literature. For each promoter class, we calculated the percentage of promoters containing H2A.Z nucleosomes. Turnover rates of histone H3 were taken from Dion et al. [27], which were normalized among all promoters in that turnover rates at promoters are higher than those in coding region [27], such that their means are zero and standard deviations are one. The data of sequence preferences for nucleosomes were taken from Segal et al. [36] and Lee et al. [34], which were normalized, such that their values are between 0 and 1. We compared the intrinsic DNA sequence preferences among the three cohorts by considering both the top ten maximal preferences values (at least 20 bp interval each other) and the mean value at each promoter.

### Binding sites

Transcription factor binding sites were taken from Harbison et al. [37], which includes the binding sites of 203 TFs at promoters in YPD medium. A *P *value cutoff of 0.001 was used to define the set of genes bound by a particular TF. For every subset of promoters considered in the main text, we calculated the percentage of promoters having multiple transcription factor binding sites, and the percentage of binding sites localized in nucleosome [34]. TATA-containing genes were taken from Basehoar et al. [41]. RNAP II occupancy data were taken from Steinmetz et al. [44], which were normalized among all promoters (200 bp upstream regions), such that their means are zero and standard deviations are one.

### Gene expression

The transcription rates and mRNA abundance were taken from Holstege et al. [26], which were normalized, such that their means are zero and standard deviations are one. We compiled available gene expression data from the Stanford Microarray Database [47], a total of 1,082 published microarray experiments for 6,260 genes in various cellular conditions. For each gene, we calculated the proportion of experiments in which it displayed significantly up-regulated expression changes, and defined the normalized resulting value as openING rate. To avoid confusion due to experimental noise, we set a relatively strict threshold (2.5-fold) for significantly up-regulated expression changes.

### Other data

Genome-wide expression data accompanying the mutation of some specific lysines (H3K4, H3K27, H3N-terminal, H4K8, H4K12, H4K16, and H4N-terminal) were taken from Martin et al. [32] and Dion et al. [33], respectively. Genome-wide expression data corresponding to the deletion of various TFs were taken from Hu et al. [24]. Protein-protein interactions data (2.0.44 version of Saccharomyces cerevisiae) were taken from BioGRID [31], a database of protein and genetic interactions from major model organism species.

## Authors' contributions

ZD and XD analyzed the results and drafted the manuscript, and ZD also designed the study, implemented the algorithms, carried out the experiments. QX, JF, YD, JW and CH participated in the analysis and discussion. All authors read and approved the final manuscript.

## Supplementary Material

Additional file 1 **Table S1**. ORF names for the modification cohort genes, positive and negative remodeling cohort genes, targets of remodelers. Click here for file

Additional file 2 **Table S2**. The interactions between chromatin remodeling and histone modifications. Click here for file

Additional file 3 **Table S3**. The interactions between chromatin remodeling and histone modifications using remodeler occupancy data. Click here for file

Additional file 4 **Table S4**. ORF names for the modification-independent cohort genes, remodeling-independent cohort genes, and modification and remodeling cohort genes. Click here for file

Additional file 5 **Figure S5**. Modification levels and gene expression levels accompanying the perturbation of chromatin remodelers for the three gene cohortsMaximum level among the 25 modifications (y-axis) and maximum expression level (log2 transformed and the absolute value taken) accompanying the perturbation of 33 chromatin remodelers (x-axis), are plotted for each gene in modification-independent cohort, remodeling-independent cohort, and modification and remodeling cohort, respectively. Genes assigned to each of the three cohorts are significantly different from each other. Click here for file

Additional file 6 **Table S6**. The K-S P values that evaluate difference in the distribution of gene expression values accompanying the mutation of TFs between the modification cohort genes and the rest of the genes. Click here for file

Additional file 7 **Table S7**. Significance values relating genomic properties to the three cohorts P values for Gene Ontology terms were derived using 'GO term finder' at the Saccharomyces Genome Database. Click here for file
