# Supplementary figures and images for: Genome-wide analysis of interactions between ATP-dependent chromatin remodeling and histone modifications
Source: BMC Genomics. 2009 Jul 8;10:304. doi: 10.1186/1471-2164-10-304 (PMC2713269; doi:10.1186/1471-2164-10-304)

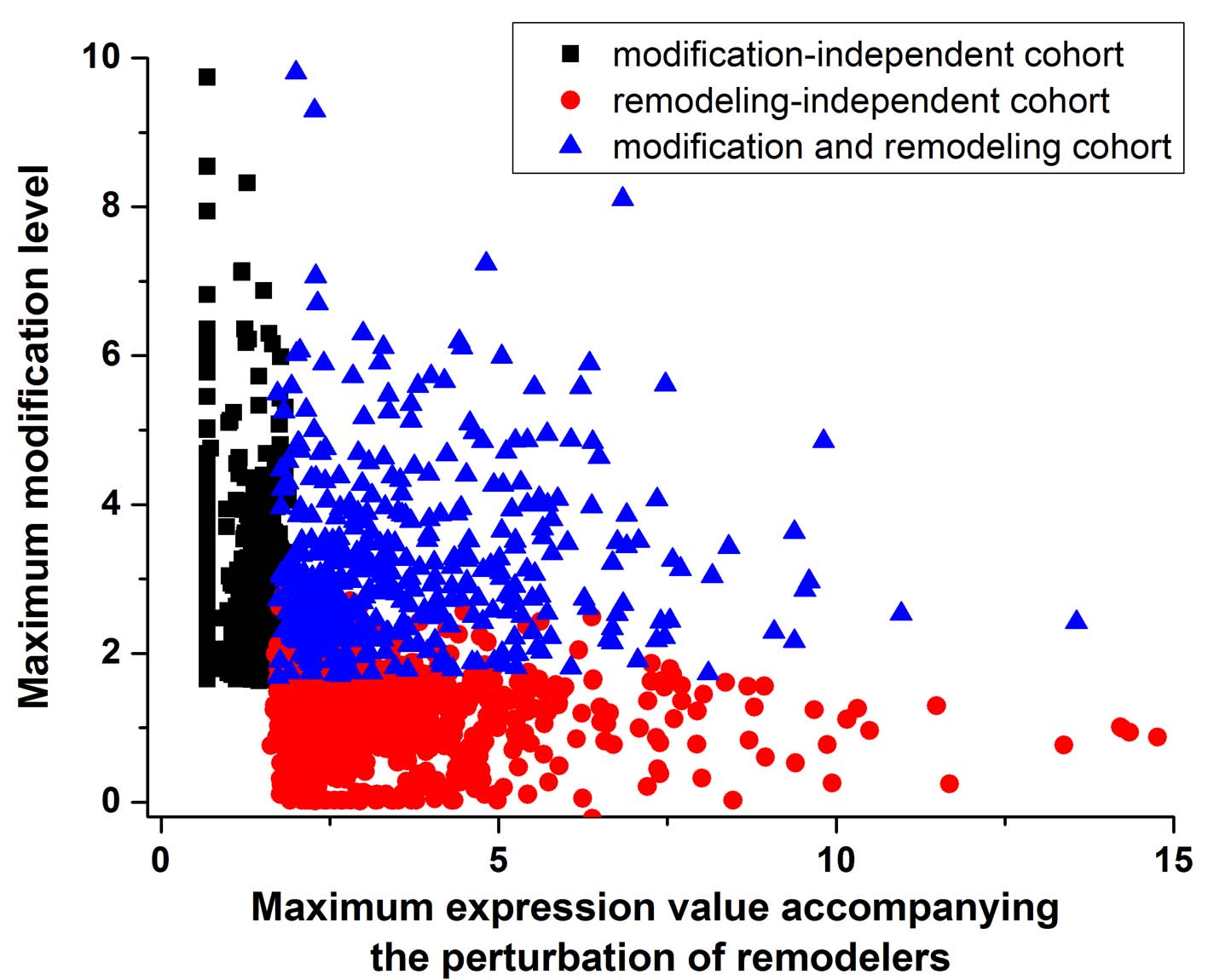

Supplement: Additional file 5 — Figure S5. Modification levels and gene expression levels accompanying the perturbation of chromatin remodelers for the three gene cohortsMaximum level among the 25 modifications (y-axis) and maximum expression level (log2 transformed and the absolute value taken) accompanying the perturbation of 33 chromatin remodelers (x-axis), are plotted for each gene in modification-independent cohort, remodeling-independent cohort, and modification and remodeling cohort, respectively. Genes assigned to each of the three cohorts are significantly different from each other. [file 1471-2164-10-304-S5.jpeg]
